# Role of inflammation in initiation and maintenance of atrial fibrillation in rheumatic mitral stenosis – An analytical cross‐sectional study

**DOI:** 10.1002/joa3.12428

**Published:** 2020-09-04

**Authors:** Gautam Sharma, Nirmal Ghati, Mohd Sharique, Shruti Sharma, Sudhir Shetkar, Suman Karmakar, Nitish Naik, Ramakrishnan Lakshmy, Bhaskar Thakur, Aman Agarwal, Anita Saxena

**Affiliations:** ^1^ Department of Cardiology All India Institute of Medical Sciences New Delhi India; ^2^ Department of Pathology, National Institute of Pathology, Indian Council of Medical Research, Safdarjung Hospital campus New Delhi India; ^3^ Department of Cardiac Biochemistry, All India Institute of Medical Sciences New Delhi India; ^4^ Department of Biostatistics All India Institute of Medical Sciences New Delhi India; ^5^ Centre for Integrative Medicine and Research All India Institute of Medical Sciences New Delhi India

**Keywords:** atrial fibrillation, inflammation, mitral stenosis, rheumatic heart disease, serum biomarkers

## Abstract

**Background:**

Inflammation has been implicated in the initiation and perpetuation of non‐valvular atrial fibrillation (AF). However, there is a lack of similar data on AF in rheumatic heart disease (RHD). The objective of this study was to analyze the association of inflammation as measured by serum inflammatory biomarkers with AF in rheumatic mitral stenosis (Rh‐MS).

**Methods:**

A comparative cross‐sectional analytical study was conducted on 181 Rh‐MS patients in normal sinus rhythm (NSR; n = 69), subclinical transient AF (SCAF; detected by 24‐hours Holter monitoring; n = 30) and chronic AF (n = 82). Serum hs‐CRP, IL‐6, and sCD‐40L were assessed using ELISA immunoassay and compared in all groups of Rh‐MS with or without AF.

**Results:**

We found significantly higher serum hs‐CRP and sCD‐40L levels in the overall AF (Chronic AF + SCAF) group (*hs‐CRP*: 4.5 ± 3.4 vs 2.3 ± 2.9 mg/L, *P* < .01; *sCD‐40L*: 6.4 ± 4.8 vs 3.1 ± 3.4 ng/mL, *P* < .01) and chronic AF subgroup (*hs‐CRP*: 4.9 ± 3.4 vs 2.3 ± 2.9 mg/L, *P* < .01; *sCD‐40L*: 6.9 ± 5.1 vs 3.1 ± 3.4 ng/mL, *P* < .01) compared to patients with sinus rhythm. There was a statistically significant graded increase of serum IL‐6 level from the NSR to the SCAF (*vs NSR*: 6.8 ± 3.9 vs 4.0 ± 2.2 pg/mL, *P* = .03), and chronic AF subgroups (*vs NSR*: 9.3 ± 6.5 vs 4.0 ± 2.2 pg/mL, *P* < .01; vs *SCAF*: 9.3 ± 6.5 vs 6.8 ± 3.9, *P* = .05) of atrial fibrillation.

**Conclusions:**

Elevated levels of serum hs‐CRP, IL‐6, and sCD‐40L were strongly associated with overall AF and also with SCAF and chronic AF in Rh‐MS patients indicating a potential role of inflammation in the pathophysiology of rheumatic AF.

## Graphical abstract

- Progressive increase was observed in biomarkers level in NSR, SCAF, and chronic AF.
- Elevated inflammation among three groups may represent progressive stages in rheumatic AF pathophysiology.
- Low‐grade inflammation is associated with AF in RHD patients.
- Graded biomarkers level shows increasing AF burden with higher inflammation.
- Patients can be intensively screened for prophylactic stroke preventive management.

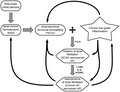

## 1 INTRODUCTION

Pathophysiology of atrial fibrillation (AF) is highly complex and involves both structural and electrical remodelling of the left atrium. The role of inflammation in initiating and propagating non‐valvular atrial fibrillation has been suggested in several studies using inflammatory biomarkers [like high‐sensitivity C‐reactive protein (hs‐CRP), Interleukin‐6 (IL‐6), Soluble CD‐40 Ligand (sCD‐40L)] as a measure of systemic inflammation.^1^, ^2^, ^3^, ^4^, ^5^, ^6^, ^7^, ^8^, ^9^ However, it is dilemmatic whether chronic inflammation is a risk factor or a consequence of AF.^10^ Bruins et al,^11^ for the first time, showed a link between inflammation and postoperative non‐valvular AF in patients undergoing coronary artery bypass surgery. However, such an association has not been explored widely in patients with rheumatic heart disease (RHD), which is commonly encountered in low‐ and middle‐income group countries. Atrial fibrillation in rheumatic heart disease affects a younger population, is influenced by factors different from those affecting non‐valvular atrial fibrillation and has a higher risk of thromboembolism. Previous histopathological studies have shown degenerative remodelling, extensive fibrosis, and evidence of ongoing inflammation in RHD patients with AF, thereby suggesting an association between myocardial inflammation and AF.^12^ A previous serum biomarker study also showed statistically higher levels of hs‐CRP and IL‐6 in RHD patients with paroxysmal and permanent AF.^13^ However, all these studies have a small sample size, and their findings have not been validated in larger population. Furthermore, there is a need for non‐invasive tests which may allow detection of low‐grade inflammation in RHD patients and thus help in identifying patients predisposed to the development of atrial fibrillation.

In the present research, we sought to study the role of inflammation by comparing the levels of inflammatory biomarkers (hs‐CRP, IL‐6, and sCD‐40L) in rheumatic mitral stenosis (Rh‐MS) patients with and without AF. Additionally, the association of various echocardiographic and demographic parameters as risk factors for atrial fibrillation was also studied.

## 2 METHODS

### 2.1 Study design

The comparative cross‐sectional analytical study was conducted in a tertiary care referral hospital in New Delhi, India. The clinical study was conducted in accordance with the ethical standards of the institutional committee on human experimentation and with the Helsinki Declaration of 1975, as revised in 2000. The study was approved by the institutional ethics committee. The study duration was three years, and written informed consent was obtained from all eligible patients.

### 2.2 Study participants

Patients aged between 18 and 45 years with isolated or predominant Rh‐MS (mitral valve area [MVA] <2 cm^2^ on echocardiography) were included. The patients were excluded if there were any confounding factors that could result in a higher incidence of AF or elevated levels of inflammatory biomarkers. Patients with left ventricular (LV) systolic dysfunction (ejection fraction [EF] < 50%), coronary artery disease, hypertension (blood pressure > 140/90 mm Hg or receiving anti‐hypertensive), recent cerebrovascular accidents (within past three months), hyperthyroidism, diabetes mellitus (fasting blood sugar > 126 mg/dL or receiving oral hypoglycaemia drugs/insulin), obesity (body mass index[BMI] > 30 kg/m^2^), acute rheumatic activity, any acute or chronic infections, chronic inflammatory diseases, malignancy, liver dysfunction, chronic obstructive pulmonary disease, recent major surgical procedure (within past three months), renal failure (serum creatinine > 2.5 mg/dL or on dialysis), and any previous surgical or percutaneous intervention on the mitral valve were excluded.

### 2.3 Patient recruitment and assessment

Baseline demographic data and clinically relevant history (including intake of oral anticoagulants [OAC]) were obtained from all the eligible participants. All patients underwent investigations including electrocardiogram (ECG), complete hemogram, erythrocyte sedimentation rate, fasting blood sugar, urea, creatinine, electrolytes, bilirubin, and transaminases. The patients who had baseline atrial fibrillation during the initial assessment were re‐evaluated by electrocardiography at one‐month gap to confirm the chronicity of atrial fibrillation. A detailed transthoracic echocardiographic examination (***iE33; Philips Medical Systems, Bothell, Washington***) was performed using a standard protocol. MVA was calculated by continuous‐wave Doppler by pressure half‐time as well as by 2D planimetry methods. Patients with MVA < 1 cm^2^ were classified as severe MS, MVA 1.0 to 1.5 cm^2^ as moderate MS and MVA between 1.5 and 2.0 cm^2^ as mild MS. Left atrial (LA) end‐systolic diameter was measured in the parasternal long‐axis view in M‐mode. Antero‐posterior, medio‐lateral and apico‐basal diameters of the left atrium (LA) were used to calculate LA volume by the prolate ellipse method (the product of these three dimensions is multiplied with the constant value of 0.523).^14^ Wilkins scoring was evaluated at the time of echocardiography by assessing leaflet thickening, mobility, calcification, and subvalvular involvement on a scale of 0 to 4. The final score was calculated out of maximum score of 16. Left atrial and left atrial appendage clot was diagnosed when an echo‐dense mass with an echocardiographic appearance different from the atrial endocardium and the pectinate muscles was detected in at least two orthogonal views. The presence of spontaneous echo‐contrast (SEC) was diagnosed when dynamic and swirling intracavitary smoke‐like echoes were detected, which were differentiated from white noise artefact by their characteristic swirling pattern and by careful attention to the gain settings. The severity of atrial SEC was scored as, 0 = no SEC, grade 1+ = mild SEC at some part of LA, grade 2 + = severe swirling SEC that appeared throughout the atrium.^15^

**FIGURE 1:**
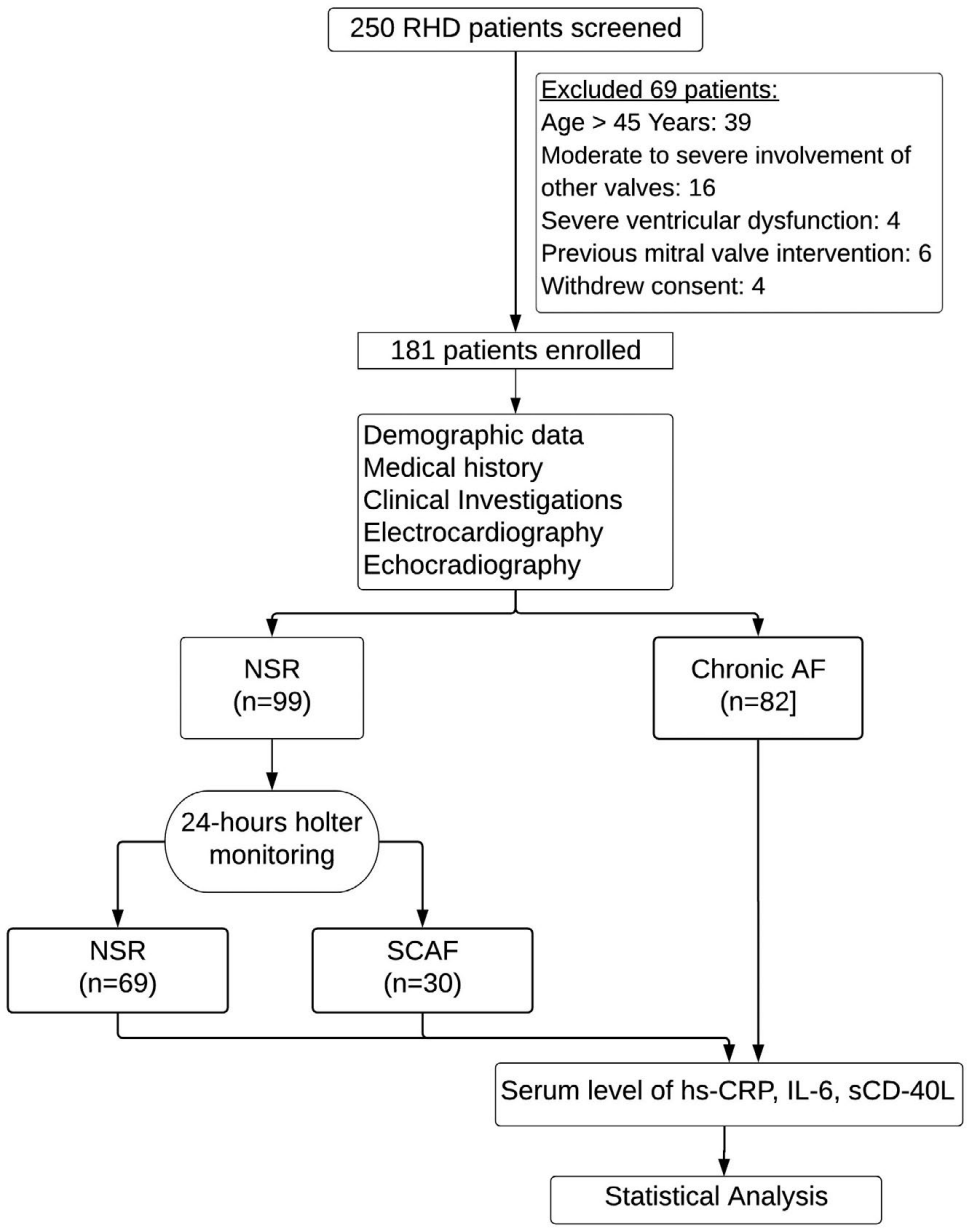
Flow diagram showing screening and recruitment of the study population. AF = atrial fibrillation, NSR = normal sinus rhythm, SCAF = subclinical transient AF, RHD = rheumatic heart disease, hs‐CRP = high sensitivity C‐reactive protein, IL‐6 = interlukin‐6, sCD‐40L = soluble CD‐40 Ligand

All the patients who were in sinus rhythm underwent a 24‐hour ECG recording using 10‐lead Holter system (***Schiller, Belmont, Australia***) to detect subclinical transient atrial fibrillation (SCAF). Subclinical transient AF is defined as bursts of irregular tachycardia consisting of more than three consecutive QRS complexes with a rate of >100 beats per minute with no discernible P waves.^16^, ^17^ Based on the Holter analysis (Table S1), the patients were categorized in the NSR group (n = 69) and the SCAF group (n = 30). The third group comprised patients with chronic AF (n = 82) (Figure 1).

### 2.4 Measurements of inflammatory biomarkers

Biomarkers of inflammation—that is, high sensitivity C‐reactive protein (hs‐CRP), Interleukin‐6 (IL‐6) and soluble CD‐40 Ligand (sCD‐40L) were measured by standard ELISA immunoassay kits for hs‐CRP (***BioCheck, Inc Foster City, USA***), IL‐6 (***Quantikine Elisa, R & D Systems, Minneapolis, USA***), and sCD‐40L (***Diaclone SAS, Besancon Cedex, France***). As ELISA kits have high fidelity and results may vary between two same ELISA kits from the same or two different manufacturers, all these samples were run along with the quality control (QC) with already defined analysis range to check the accuracy and authenticity of the tests. Additionally, 20 age‐matched healthy subjects were added to assess the performance of the biomarkers.

### 2.5 Statistical analysis

Continuous variables were expressed as mean ± standard deviation (SD) or median (range). The frequency of categorical variables was expressed as percentage (%). Independent t‐test was used to compare the normally distributed continuous variable between two groups. Chi‐square test was used to analyze the association between two categorical variables. The diagnostic accuracy of the significant inflammatory biomarkers, demographic, and echocardiographic parameters were assessed using the receiver operating characteristic curve analysis. Univariate binary logistic regression was performed to calculate the unadjusted odds ratio, whereas, multiple logistic regression analysis was performed to determine confounding variables adjusted odds ratios. A two‐sided *P* ≤ .05 was considered significant. Statistical software Stata 14 (***Stata Corp, 4905 Lakeway Drive, Texas, USA***) was used for statistical analysis.

## 3 RESULTS

A total of 250 RHD patients attending the cardiology out‐patient clinic were screened for inclusion, and after initial evaluation and echocardiography, 69 patients were excluded (39 patients were above 45 years, 16 patients had moderate to severe involvement of other valve lesions, four patients had severe ventricular dysfunction, six patients previously underwent mitral valve interventions, and four patients withdrew informed consent) (Figure 1).

### 3.1 Study population characteristics

The characteristics of the study population (n = 181) and its group‐wise description is shown in Table 1. Among all the Rh‐MS patients, 45.3% of patients had chronic atrial fibrillation, and 16.6% of patients had subclinical transient atrial fibrillation. Mean age ± SD of the healthy subjects, NSR, overall AF, SCAF, and chronic AF groups were 28.6 ± 2.6, 29.3 ± 7.2, 34.6 ± 7.1, 33.3 ± 7.7, and 35.1 ± 6.9 years, respectively. A total of 66 (80.5%) out of 82 patients with chronic AF was on oral anticoagulation therapy. History of stroke was present in only three patients in the chronic AF subgroup.

**TABLE 1:** Comparison of the demography, echocardiographic parameters, and serum inflammatory biomarker levels across the different study groups of Rh‐MS patients

Parameters	Sinus rhythm	Overall AF^a^		Subclinical transient AF		Chronic AF		
	(n = 69)	(n = 112)	*P* ^b^	(n = 30)	*P* ^b^	(n = 82)	*P* ^b^	*P* ^c^
Age (Years)	29.3 ± 7.2	34.6 ± 7.1	**<.01**	33.3 ± 7.7	**.01**	35.1 ± 6.9	**<.01**	.25
Male	43.5	45.5	.78	46.7	.76	45.1	.84	.88
BMI	19.1 ± 3	19.4 ± 3	.4	20.1 ± 3	.12	19.2 ± 2.8	.77	.15
Dyspnoea (NYHA Class)								
I	2.9	0.9	.37	0	.52	1.1	.42	.67
II	69.6	60.7		63.3		59.8		
III	26.1	35.7		36.7		35.4		
IV	1.4	2.7		0		3.7		
MVA (cm^2^)	0.9 ± 0.4	0.8 ± 0.2	**.03**	0.7 ± 0.2	.12	0.8 ± 0.2	.09	.49
MDG (mmHg)	12.9 ± 6.1 (n* = 62*)	12.6 ± 4.4 (n* = 108*)	.7	12.5 ± 5.7 (n* = 28*)	.78	12.6 ± 3.9 (n* = 80*)	.73	.91
Severity of MS								
Mild	7.2	0	**<.01**	0	.25	0	**<.01**	.32
Moderate	17.4	8.9		13.3		7.3		
Severe	75.4	91.1		86.7		92.7		
LA volume (ml)	150.8 ± 62.2	151.9 ± 49.7	.89	136.8 ± 44.3	.26	157.4 ± 50.7	.47	.05
LA Volume indexed (ml/m^2^)	102.8 ± 44.6	100.2 ± 32.9	.65	89.2 ± 29.4	.13	104.2 ± 33.3	.81	.03
Wilkins Score	7.1 ± 1.8	8.0 ± 1.8	**<.01**	8.2 ± 1.8	**<.01**	7.9 ± 1.8	**.01**	.43
LA/LAA clot	0	17.9	**<.01**	3.3	.30	23.2	**<.01**	.01
SEC								
Grade 0	4.3	3.6	.17	3.3	.19	3.7	.18	.65
Grade 1+	2.9	2.7		0		3.7		
Grade 2+	0	5.4		3.3		6.1		
RVSP (mmHg)	57.7 ± 23.7 (n* = 39*)	50.9 ± 19.4 (n* = 80*)	.1	58.9 ± 24.6 (n* = 14*)	.86	49.2 ± 17.9 (n* = 66*)	**.04**	.08
LVEF (%)	60.0 ± 2.8	59.3 ± 2.6	.08	60 ± 0	.99	59.0 ± 3.0	**.04**	.08
hs‐CRP (mg/l)	2.3 ± 2.9	4.5 ± 3.4	**<.01**	3.4 ± 3.1	.34	4.9 ± 3.4	**<.01**	.09
IL‐6 (pg/ml)	4.0 ± 2.2	8.6 ± 6.0	**<.01**	6.8 ± 3.9	**.03**	9.3 ± 6.5	**<.01**	**.05**
sCD‐40L (ng/ml)	3.1 ± 3.4	6.4 ± 4.8	**<.01**	5.2 ± 3.8	.07	6.9 ± 5.1	**<.01**	.20

Note

Values are mean ± SD or (%); Bold values are significant (*P* ≤ .05)

Abbreviations: AF, atrial fibrillation; Rh‐MS, rheumatic mitral stenosis; SD, standard deviation; NYHA, New York Heart Association; MVA, mitral valve area; MDG, mean diastolic gradient; MS, mitral stenosis; BMI, body mass index; SEC, spontaneous echo contrast; LVEF, left ventricular ejection fraction; RVSP, right ventricular systolic pressure; LA, left atrium; LAA, left atrial appendage; hs‐CRP, high sensitivity C‐reactive protein; IL‐6, interleukin‐6; sCD‐40L, soluble CD‐40 Ligand. SI unit: ng (nanogram); l(liter); mg(milligram); ml(milliliter); pg(picogram).

^a^ Overall AF: Subclinical transient AF + chronic AF

^b^ vs sinus rhythm.

^c^ vs SCAF.

### 3.2 Results from univariate analysis

**FIGURE 2:**
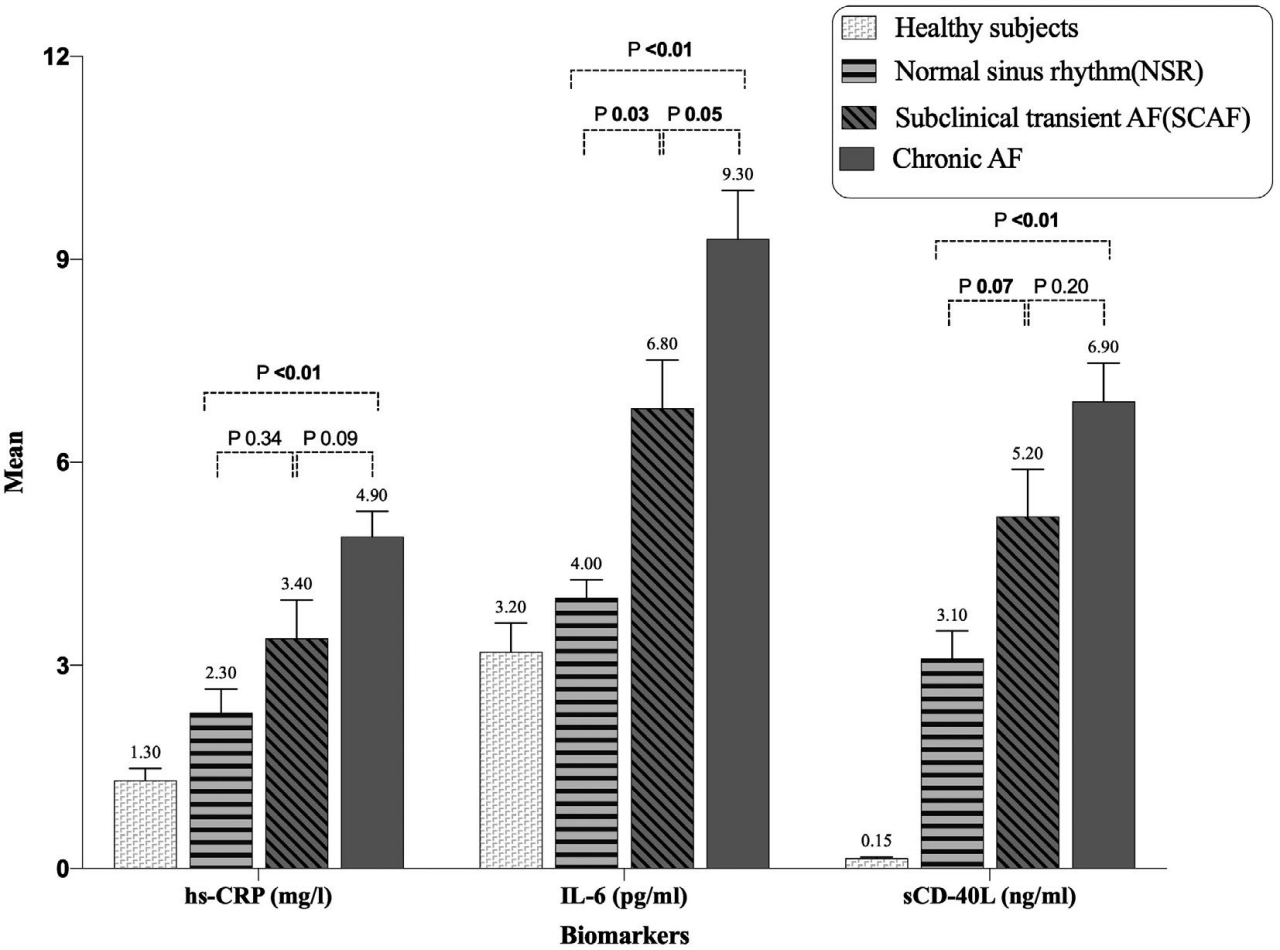
Serum inflammatory biomarkers levels in healthy subjects and across various rheumatic AF groups. AF: atrial fibrillation, hs‐CRP: high sensitivity C‐reactive protein, IL‐6: interleukin‐6, sCD‐40L: soluble CD‐40 Ligand. SI unit: mg (milligram); pg (picogram); ng (nanogram); l(liter); ml(milliliter)

Significantly higher number of patients with severe MS had AF. In addition, Rh‐MS patients with AF had significantly higher Wilkins score and lower MVA in comparison to the NSR group. The serum hs‐CRP, IL‐6, and sCD‐40L levels (mean ± SD) in the healthy subjects were 1.3 ± 0.8 mg/L, 3.2 ± 1.9 pg/mL, and 0.1 ± 0.1 ng/mL, respectively. Serum inflammatory biomarker (hs‐CRP, IL‐6, sCD‐40L) levels were significantly higher in the Rh‐MS patients with AF in comparison to the NSR group (Table 1). In addition, there was a graded increase in the levels of the inflammatory biomarkers across the healthy subjects, NSR and various AF subgroups (Figure 2 **)**.The levels of significant risk factors, that is, inflammatory biomarkers (hs‐CRP, IL‐6, sCD‐40L), age, MVA, Wilkins score were dichotomized for the purpose of regression analysis, using the cut‐offs values with optimum sensitivity and specificity derived by constructing receiver operating characteristic curves (Table S2, S3). The area under the receiver operating characteristic (AUROC) curve, sensitivity, and specificity of the significant inflammatory biomarkers, demographic, and echocardiographic parameters were also reported in Figures S1, S2; Tables S2, S3).The comparison of the inflammatory biomarkers, demographic, and echocardiographic risk factors among the study groups is shown in Tables 1 and 2.

**TABLE 2:** Logistic regression analysis of clinical and investigational parameters showing association with atrial fibrillation in rheumatic mitral stenosis patients

Parameters	Univariate regression analysis			Multiple regression analysis		
	Odd ratio	95% confidence interval	*P*	Odd ratio	95% confidence interval	*P*
Age ≥ 32 yr	3.9	2.1‐7.4	**<.01**	6.6	2.7‐16.5	**<.01**
Severe MS	3.2	1.5‐6.9	**<.01**	5.0	1.4‐18.2	**.01**
MVA ≤ 0.8 cm^2^	1.0	0.5‐1.8	.91	0.5	0.2‐1.3	.15
Wilkins score ≥ 8	3.3	1.7‐6.2	**<.01**	3.2	1.3‐7.8	**.01**
hs‐CRP ≥ 2.02 mg/l	5.8	3.0‐11.3	**<.01**	4.2	1.7‐10	**<.01**
IL‐6 ≥ 4.92 pg/ml	6.7	3.4‐13.1	**<.01**	7.0	2.8‐17.7	**<.01**
sCD‐40L ≥ 3.1 ng/ml	4.7	2.5‐9.0	**<.01**	5.5	2.2‐13.4	**<.01**

Note

Bold values are significant (*P* ≤ .05).

hs‐CRP, high sensitivity C‐reactive protein; IL‐6, interleukin‐6; sCD‐40L, soluble CD‐40 Ligand; MS, mitral stenosis; MVA, mitral valve area; SI unit: ng (nanogram); pg (picogram); mg (milligram); ml (milliliter); l (liter)

### 3.3 Results from multivariable analysis

Age ≥ 32 years [Odd Ratio 6.6 (95% Confidence interval 2.7‐16.5); *P* < .01], severe MS [5 (95% CI 1.4‐18.2); *P* = .01], Wilkins score ≥ 8 [3.2 (95% 1.3‐7.8); *P* = .01],

hs‐CRP ≥ 2.02 mg/L [4.2 (95% CI 1.7‐10); *P* < .01], IL‐6 ≥ 4.92 pg/mL [7 (95% CI 2.8‐17.7); *P* < .01], and sCD‐40L ≥ 3.10 ng/mL [5.5 (95% CI 2.2‐13.4); *P* < .01] were the significant parameters that showed an independent and strong association with atrial fibrillation (Table 2) in comparison to the sinus rhythm in Rh‐MS patients. Though MVA was significantly lower in the overall AF group than the NSR group, it did not show a significant association in the regression analysis.

## 4 DISCUSSION

Several risk factors, that is, age, hypertension, obesity, heart failure, alcohol intake, and family history, etc have been established as predictors of non‐valvular atrial fibrillation. However, there is a paucity of information on the risk factors associated with AF in RHD patients. Age is the most commonly reported risk factor of AF in RHD.^18^, ^19^ Our study has similarly shown a significant association of increasing age with increasing severity of atrial fibrillation. There was also an independent association with overall AF in the multiple regression analysis.

Among the echocardiographic parameters, mitral valve area (MVA), mean diastolic gradient (MDG), left atrial diameters, left atrial volume, and volume index are the most commonly reported risk factors and predictors of chronic atrial fibrillation in RHD patients.^13^, ^18^, ^20^ The stenotic or regurgitant mitral valve leads to increased hemodynamic stress resulting in left atrial cell damage, fibrosis, and enlargement influencing the conduction properties. This eventually initiates and maintains a self‐propagating cycle of chronic atrial fibrillation.^12^, ^18^, ^20^ In our study, the presence of severe mitral stenosis (ie, MVA < 1 cm^2^) and higher Wilkins score were the significant echocardiographic parameters associated with atrial fibrillation. MS severity and Wilkins score ≥ 8 also showed an independent association with atrial fibrillation in multiple regression analysis. In addition, patients with chronic AF had more severe MS and higher Wilkins scores than patients with sinus rhythm. Altogether, these findings indicate that chronically elevated left atrial hemodynamic pressure due to lower mitral valve area and sub‐valvular disease is one of the main pathogenic factors for initiation and perpetuation of atrial fibrillation in Rh‐MS patients. Contrary to the earlier studies on non‐valvular and valvular AF patients,^13^, ^18^, ^20^ the left atrial dimension did not show any significance. It may be postulated that in valvular AF, left atrial enlargement is a consequence of rheumatic activity rather than a consequence of AF.

Chronic low‐grade inflammation is one of the important risk factors for the initiation and perpetuation of non‐valvular atrial fibrillation. The strong association between inflammation and atrial fibrillation is evident from the higher frequency of atrial fibrillation in patients with pericarditis and myocarditis. Most of the clinical studies analyzed the presence of low‐grade inflammation in AF patients by either histopathological examination of a left atrial appendage or by measuring serum inflammatory biomarkers. The most commonly tested serum biomarker for this purpose is C‐reactive protein (CRP). It is a serum soluble acute‐phase protein (APP) produced by the hepatocytes in response to cytokines (IL‐6 and IL‐1) during inflammation elsewhere in the body.^21^ It has been hypothesized that CRP may attach to the atrial myocyte and initiate a complement‐mediated local inflammation contributing to the pathophysiology of atrial fibrillation.^22^, ^23^ Several studies have reported a higher serum CRP level in patients with atrial fibrillation in comparison to control subjects in sinus rhythm.^1^, ^2^, ^3^, ^24^, ^25^ Besides, higher CRP level has been noted in persistent AF in comparison to the paroxysmal AF, and in chronic long‐standing AF in comparison to the new onset AF.^3^, ^4^ It also has prognostic importance as the higher value of serum CRP predicted unsuccessful cardioversion of AF in several studies.^4^, ^26^, ^27^, ^28^ However, all these studies have included only non‐valvular AF patients. In our study, significantly higher serum CRP level has been noted in the overall AF and the chronic AF groups in comparison to the Rh‐MS patients with sinus rhythm. Lack of significant difference in the CRP value between the SCAF and NSR group may indicate that CRP induced local inflammation is important for transformation into and maintenance of chronic AF, not in the initial stage of subclinical AF. Relatively higher CRP value in the chronic AF group in comparison to the SCAF group further supports this hypothesis.

Interleukin 6 (IL‐6) is a serum cytokine synthesized by neutrophils and macrophages in inflammatory lesions under the influence of TNF‐α and IL‐1β. Apart from its profound pro‐inflammatory effects, IL‐6 induces the production of CRP and other acute‐phase proteins (ie, serum amyloid protein A, ferritin, fibrinogen, etc) in the liver.^29^ Several studies have reported a significantly higher level of serum IL‐6 in patients with non‐valvular AF (NVAF) in comparison to the NSR cohort.^5^, ^6^, ^30^ In our study, IL‐6 not only showed a significant association with AF in comparison to the NSR group, but also a statistically significant graded increase in the serum level in subclinical and chronic subgroups of atrial fibrillation. This finding supports a direct pro‐inflammatory effect of IL‐6 besides its indirect effects through CRP, in the initiation and maintenance of AF in rheumatic heart disease.

Our study also showed a significantly higher level of sCD‐40L in the overall AF and chronic AF groups in comparison to the NSR group. CD‐40/CD‐40L system belongs to tumour necrosis factor superfamily and has a strong pro‐inflammatory, prothrombotic, and atherogenic role.^7^ The soluble CD‐40L (sCD‐40L) has been reported to be an inflammatory marker for non‐valvular atrial fibrillation, especially during the first year of initiation.^8^ Several studies have also highlighted it as a predictor of thromboembolic complications in AF patients.^9^, ^31^ Our study is the first report that showed a significant involvement of sCD40L in the pathophysiology of atrial fibrillation in the RHD population.

**FIGURE 3:**
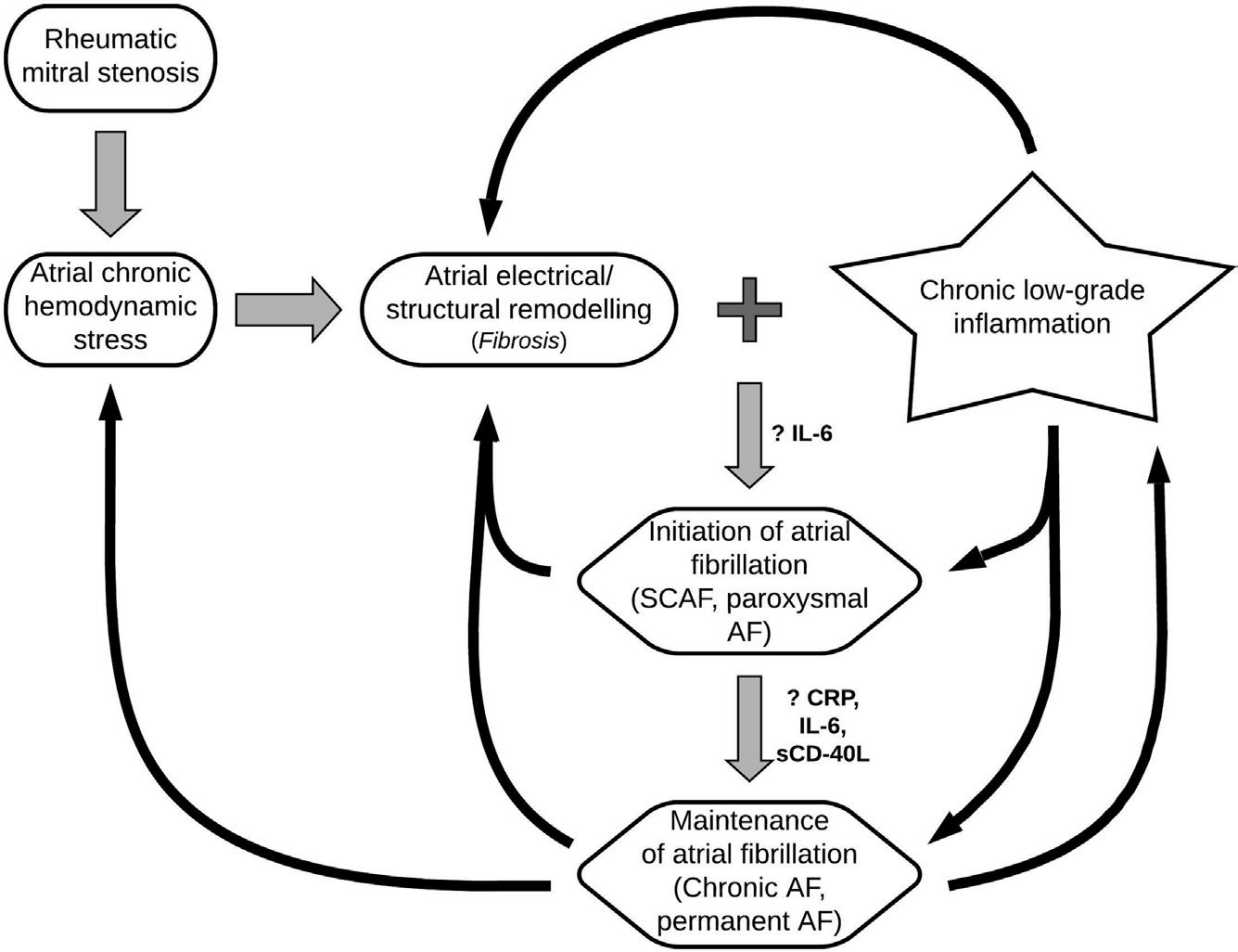
Diagram showing probable pathophysiologic mechanisms for initiation and maintenance of atrial fibrillation in rheumatic mitral stenosis. SCAF: subclinical transient atrial fibrillation, AF: atrial fibrillation, hs‐CRP: high sensitivity C‐reactive protein, IL‐6: interleukin‐6, sCD‐40L: soluble CD‐40 Ligand

Not all severe Rh‐MS patients with significant hemodynamic stress develop atrial fibrillation indicating the influence of an unknown triggering factor in the pathophysiologic cascade. It has been hypothesized that chronic inflammation may be ‘the distinctive and decisive factor’ that completes the missing link between atrial fibrillation and hemodynamic stress in rheumatic mitral stenosis. While the association of elevated levels of inflammatory biomarkers with AF does not imply causation, the presence of a graded response with increasing AF burden in our study lends merit to the hypothesis that chronic inflammation may act synergistically with hemodynamic stress to initiate and perpetuate atrial fibrillation (Figure 3). Moreover as the severity of inflammation as measured by the biomarker levels increases, the burden of AF increases (from SCAF to chronic AF) (Figure 2). The implications of such a finding would be manifold. First, these or similar inflammatory markers could be used as predictors for AF. This could be either using the marker alone or as a risk score incorporating clinical, echocardiographic, and biochemical parameters. Second, such predictors of increased risk of AF may allow upfront anticoagulation therapy in patients at risk, without overt AF, and such a strategy may help lower the burden of thromboembolic complication. Thirdly, there may be a potential role of pharmacologic immune modulators in preventing progression to AF in patients with RHD.

In this study, we have used stringent exclusion criteria to eliminate confounding factors associated with chronic inflammation that may bias the results. Although many factors like smoking, alcohol, lack of sleep, mental stress etc can induce oxidative stress, we did not collect those data which is one of the limitations of the study. Though the sample size was not ideally calculated, our observed significant results with substantial effect ensure that the study power was sufficient to detect the effect. The limitation of our study includes small sample size and cross‐sectional design. A small study group limits statistical inferences, especially in the smaller subset of patients with subclinical transient AF. Another concern is that ambulatory rhythm follow‐up of only 24 hours might have missed patients with subclinical transient and paroxysmal AF. Although there was a significant association between atrial fibrillation and serum inflammatory biomarkers (ie, hs‐CRP, IL‐6, and sCD‐40L), our study does not establish a clear cause and effect relationship between inflammation and valvular AF due to the cross‐sectional study design and small sample size. However, the potential link between chronic low‐grade inflammation and valvular AF in our study may act as a primer for further research in this area which will benefit a large subset of valvular heart disease patients in the developing countries.

## 5 CONCLUSIONS

Our study has shown a significant association between atrial fibrillation and serum inflammatory biomarkers (ie, hs‐CRP, IL‐6, and sCD‐40L), demographic (age), echocardiographic parameters (severe MS, Wilkins score, MVA) suggesting a potential synergistic role of chronic inflammation and chronic hemodynamic stress in the initiation and perpetuation of AF in rheumatic patients with predominant mitral stenosis. Furthermore, the graded increase in the serum IL‐6 level across NSR, SCAF, & chronic AF groups may represent different progressive stages in the pathophysiology of rheumatic AF. However, the potential use of the biomarkers in identifying RHD patients with predisposition to develop AF, will need further validation in a larger prospective cohort.

## Supporting information

Supplementary Material
