# Supplementary material for: Role of inflammation in initiation and maintenance of atrial fibrillation in rheumatic mitral stenosis – An analytical cross‐sectional study
Source: J Arrhythm. 2020 Sep 4;36(6):1007–15. doi: 10.1002/joa3.12428 (PMC7733567; doi:10.1002/joa3.12428)
Supplement: Supplementary file 1 — Supplementary Material [file JOA3-36-1007-s001.docx]

**Role of Inflammation in Initiation and Maintenance of Atrial Fibrillation in Rheumatic Mitral Stenosis – An Analytical Cross-Sectional Study**

**Supplementary Materials**

**Supplemental table S1**: Results of 24-hr Holter analysis in 99 Rh-MS patients with baseline sinus rhythm in the ECG.

| Findings | No of patients^†^  [n (%)] | Number of episodes |
| --- | --- | --- |
| Normal sinus rhythm | 69 (69.7) | - |
| Isolated SVE | 61 (61.6) | NA |
| SV couplets | 22 (22.2) | NA |
| SCAF | 30 (30.3) | 72 |
| Paroxysmal AF | 0 (0) | - |
| EAT | 1 (1) ‡ | 2 |
| PSVT | 1 (1) ‡ | 1 |
| NSVT | 0 (0) | - |
| Sustained VT | 0 (0) | - |

**^†^**: Total number of patients = 99

**^‡^**: These patients also had SCAF

SVE: supraventricular ectopic, SV: supraventricular, SCAF: subclinical transient atrial fibrillation, AF: atrial fibrillation, EAT: ectopic atrial tachycardia, PSVT: paroxysmal supraventricular tachycardia, NSVT: nonsustained ventricular tachycardia, VT: ventricular tachycardia, NA: not available


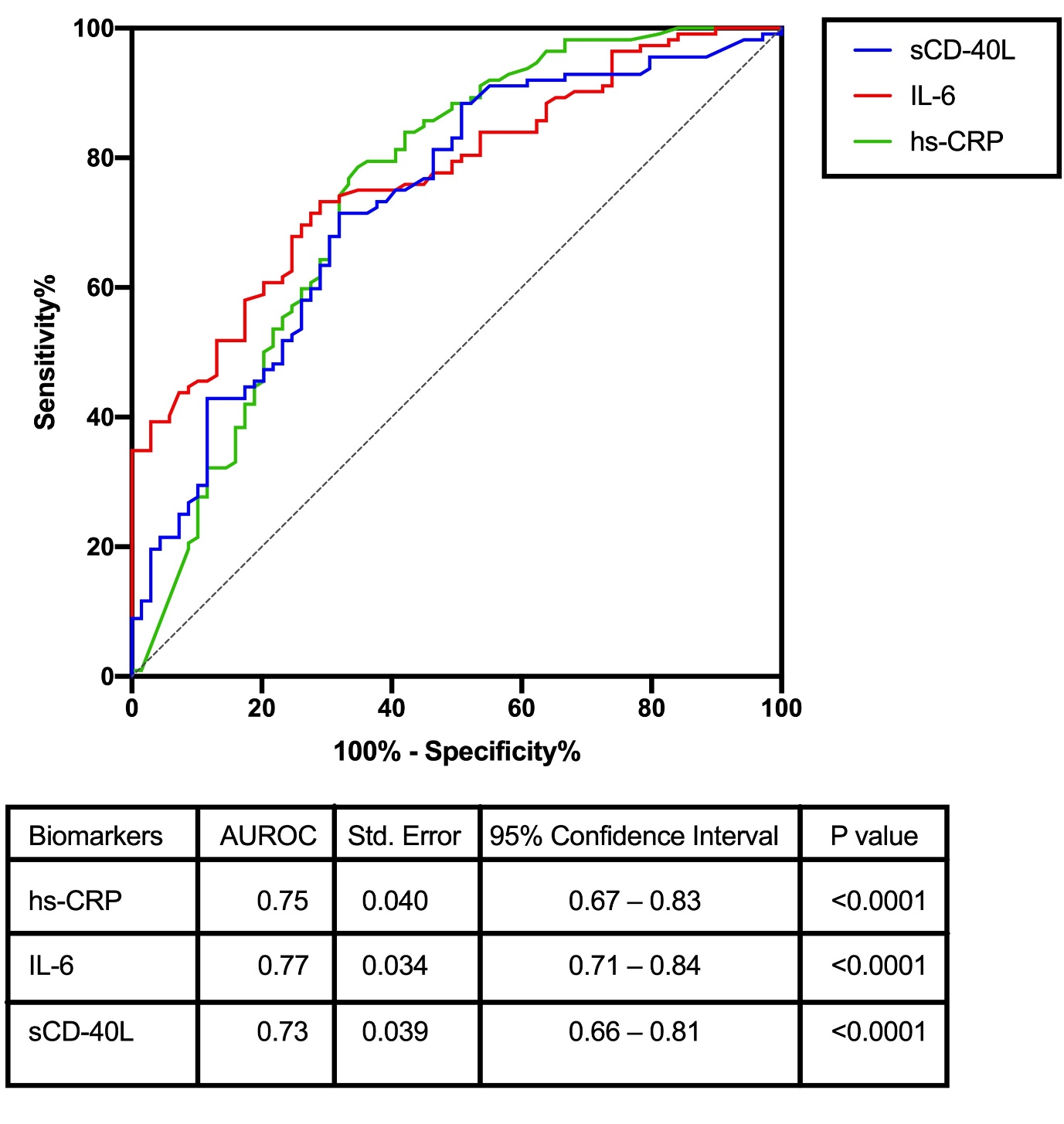


**Supplemental figure S1**: Receiver operating curve (ROC) showing diagnostic accuracy of serum inflammatory biomarkers for assessment of AF in rheumatic mitral stenosis.

AF: atrial fibrillation, AUROC: area under ROC curve, MVA: mitral valve area, MS: mitral stenosis, hs-CRP: high sensitivity C-reactive protein, IL-6: interleukin 6, sCD-40L: soluble CD-40 ligand


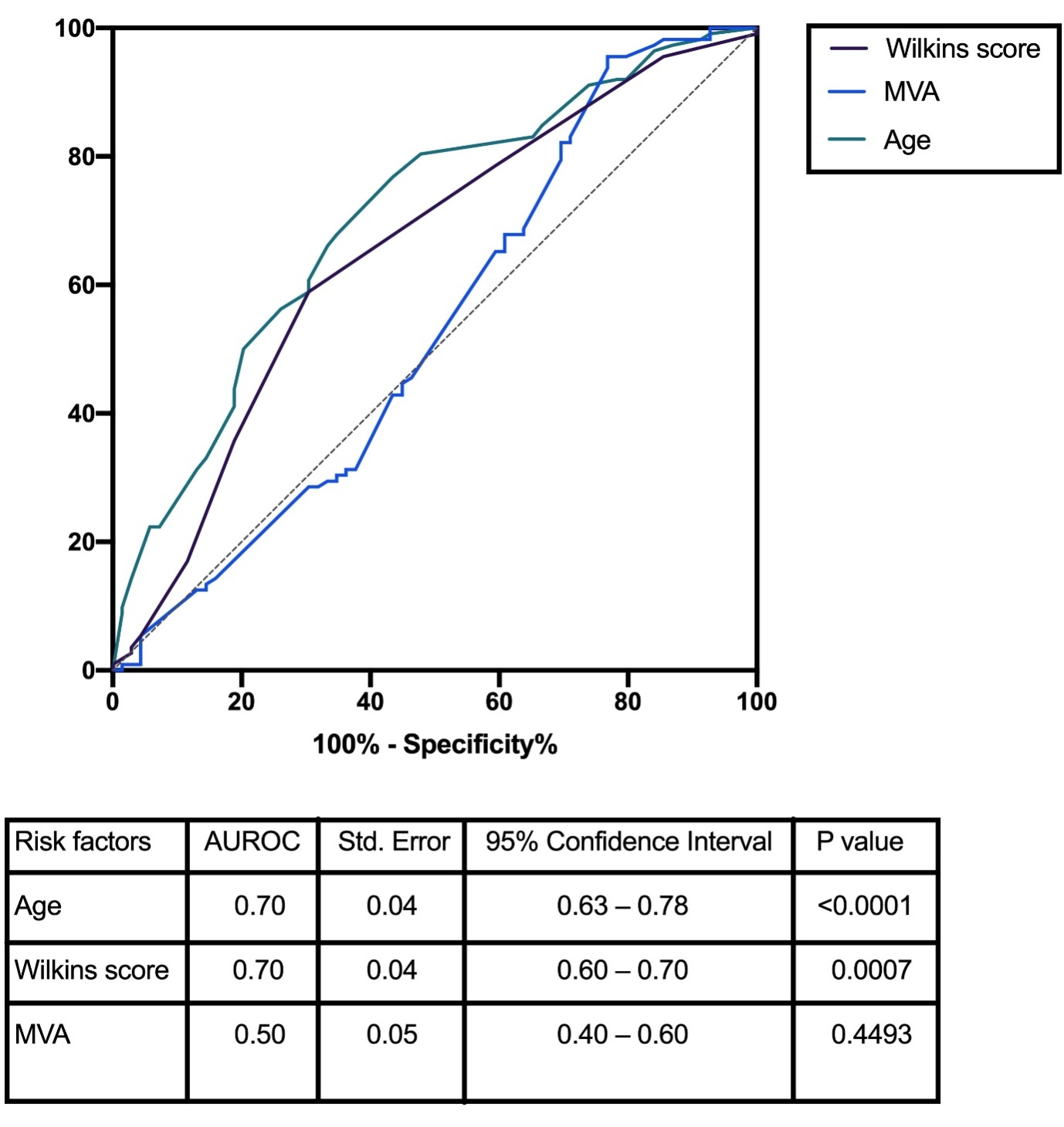


**Supplemental figure S2**: Receiver operating curve (ROC) showing diagnostic accuracy of demographic and echocardiographic parameters for assessment of AF in rheumatic mitral stenosis.

AF: atrial fibrillation, AUROC: area under ROC curve, MVA: mitral valve area, MS: mitral stenosis

**Supplemental table S2**: Sensitivity and specificity (ROC analysis) of various cutoff values of the inflammatory biomarkers for the assessment of atrial fibrillation in rheumatic mitral stenosis patients.

| **hs-CRP** | | | **IL-6** | | | **sCD-40L** | | |
| --- | --- | --- | --- | --- | --- | --- | --- | --- |
| **Cutoff**  **(mg/l)** | **Sensitivity**  **(95% CI)** | **Specificity**  **(95% CI)** | **Cutoff**  **(pg/ml)** | **Sensitivity**  **(95% CI)** | **Specificity**  **(95% CI)** | **Cutoff**  **(ng/ml)** | **Sensitivity**  **(95% CI)** | **Specificity**  **(95% CI)** |
| ≥ 1.90 | 78.57 | 65.22 | ≥ 4.87 | 74.11 | 68.12 | ≥ 2.99 | 72.32 | 62.32 |
| ≥ 1.95 | 76.79 | 66.67 | ≥ 4.88 | 73.21 | 68.12 | ≥ 3.00 | 71.43 | 63.77 |
| ≥ 2.00 | 75.89 | 66.67 | ≥ 4.90 | 73.21 | 69.57 | ≥ 3.05 | 71.43 | 66.67 |
| **≥ 2.02** | **74.11** | **68.12** | **≥ 4.92** | **73.21** | **71.01** | **≥ 3.1** | **71.43** | **68.12** |
| **≥** 2.05 | 73.21 | 68.12 | ≥ 4.99 | 71.43 | 71.01 | ≥ 3.23 | 68.75 | 68.12 |
| **≥** 2.08 | 72.32 | 68.12 | ≥ 5.00 | 71.43 | 72.46 | ≥ 3.28 | 67.86 | 68.12 |
| **≥**2.10 | 71.43 | 68.12 | ≥ 5.02 | 69.64 | 72.46 | ≥ 3.37 | 67.86 | 69.57 |

hs-CRP: high sensitivity C-reactive protein, IL-6: interleukin 6, sCD-40L: soluble CD-40 Ligand, CI-confidence interval, SI units: ng (nanogram); pg(picogram); mg(milligram); ml(millilitre); l(litre).

**Supplemental table S3**: Sensitivity and specificity (ROC analysis) of various cutoff values of the significant demographic and echocardiographic parameters for the assessment of atrial fibrillation in rheumatic mitral stenosis patients.

| **Age** | | | **MVA** | | | **Wilkins score** | | |
| --- | --- | --- | --- | --- | --- | --- | --- | --- |
| **Cutoff**  **(Years)** | **Sensitivity**  **(95% CI)** | **Specificity**  **(95% CI)** | **Cutoff**  **(cm^2^)** | **Sensitivity**  **(95% CI)** | **Specificity**  **(95% CI)** | **Cutoff** | **Sensitivity**  **(95% CI)** | **Specificity**  **(95% CI)** |
| ≥ 29 | 80.36 | 52.17 | ≥0.71 | 57.14 | 43.48 | ≥ 5 | 99.11 | 0.00 |
| ≥ 30 | 76.79 | 56.52 | ≥0.72 | 57.14 | 44.93 | ≥ 6 | 95.54 | 14.49 |
| ≥ 31 | 67.86 | 65.22 | ≥0.75 | 55.36 | 44.93 | ≥ 7 | 78.57 | 40.58 |
| ≥ **32** | **66.07** | **66.67** | ≥**0.80** | **54.46** | **46.38** | ≥**8** | **58.93** | **69.57** |
| ≥ 33 | 60.71 | 69.57 | ≥0.83 | 34.82 | 59.42 | ≥9 | 35.71 | 81.16 |
| ≥ 34 | 58.93 | 69.57 | ≥0.85 | 34.82 | 60.87 | ≥10 | 16.96 | 88.41 |
| ≥ 35 | 56.25 | 73.91 | ≥0.87 | 32.14 | 60.87 | ≥11 | 5.36 | 95.65 |

MVA-mitral valve area, CI-confidence interval, cm(centimeter)
